# Book Reviews

**Published:** 1907-02

**Authors:** 


### BOOK REVIEWS

The Care of the Teeth, by Thaddeus P. Hyatt, D. D. S., member of the Second District Dental Society of the State of New York, Brooklyn, N. Y. King Press, 1906. Price fifty cents; postage prepaid, fifty-five cents.

This little work has much to commend it. It is designed for the instruction of the laity, and is well illustrated and quite free from scientific or technical terms that would confuse the lay reader. It is written in a clear, concise style, and the author's recommendations regarding the care of the teeth, and the importance of the teeth to the comfort and physical well being of both old and young, are all good and should be influential in inducing greater attention to dental oral hygiene. We hope that it will have a large sale.

Practical Dietetics With Reference to Diet in Disease, by Alida Frances Pattee, graduate Boston Normal School of Household Arts, etc., etc. A. F. Pattee, publisher, 52 W. 39th St., New York. Price $1.00; by mail, $1.10.

This work is a practical diet book for the physician, the nurse and the patient and gives in detail the preparation of food for infants, the sick and the convalescent. It suggests appropriate food for every disease and quotes by permission the diet recommended by leading hospitals and eminent physicians in New York, Philadelphia and Boston. The book is one that should be in the hands of every physician and nurse and can well be recommended to their patients.

The Harvey Lectures. Delivered under the Auspices of the Harvey Society of New York, 1905-06. Philadelphia and London: J. B. Lippincott Company, 1906.

In the spring of 1905 there was organized in the city of New York a society the avowed object of which was the diffusion of the medical sciences by means of public lectures. The organizers of this society, which they called the Harvey Society, felt that the profession would cheerfully support an annual series of lectures dealing with the so-called experimental side of medicine given by those who devoted themselves solely to this branch of medicine. That they were correct in their surmises is evidenced by the enormous success of the first series of thirteen lectures, given under the patronage of the New York Academy of Medicine by the following distinguished experimenters: "The Theory of Narcosis," Prof. Hans Meyer; "Modern Problems of Metabolism," Prof. Carl von Noorden; "On Trypanosomes," Prof. S. G. Novy; "Autolysis," Dr. P. A. Levene; "A Critical Study of Serum Therapy," Prof. W. H. Park; "The Neurons," Prof. L. T. Barber; "Fatigue," Prof. F. S. Lee; "The Formation of Uric Acid," Prof. L. B. Mendel; "The Extent and Limitations of the Power to Regenerate in Man and Other Vertebrates," Prof. T. H. Morgan; "On the Nature and Cause of Old Age," Prof. C. S. Minot; "Modern Views Regarding Placentation," Prof. J. Clarence Webster; "Some Phases of Tuberculosis," Prof. Theobald Smith; "The Cause of Heart Beat," Prof. W. H. Howell.

The demand for this series of lectures in book form has been great, and has been gratified by the Lippincott Company in their best style. It would be difficult to conceive of a more fascinating series or one more adapted to a variety of tastes. It was a gentle tribute to the great discoverer after whom the society was named that at least one evening was devoted to a review and elaboration of his teachings by one of his modern and learned disciples.

